# Phage Therapy as an Approach to Prevent *Vibrio anguillarum* Infections in Fish Larvae Production

**DOI:** 10.1371/journal.pone.0114197

**Published:** 2014-12-02

**Authors:** Yolanda J. Silva, Liliana Costa, Carla Pereira, Cristiana Mateus, Ângela Cunha, Ricardo Calado, Newton C. M. Gomes, Miguel A. Pardo, Igor Hernandez, Adelaide Almeida

**Affiliations:** 1 CESAM and Department of Biology, University of Aveiro, Campus Universitário de Santiago, 11 3810-193 Aveiro, Portugal; 2 Food Research Division AZTI–Tecnalia, Bizkaiko Teknologi Parkea, Astondo Bidea, 609 Eraikina, 48160 Derio, Spain; Institute of Immunology and Experimental Therapy, Polish Academy of Sciences, Poland

## Abstract

Fish larvae in aquaculture have high mortality rates due to pathogenic bacteria, especially the *Vibrio* species, and ineffective prophylactic strategies. Vaccination is not feasible in larvae and antibiotics have reduced efficacy against multidrug resistant bacteria. A novel approach to controlling *Vibrio* infections in aquaculture is needed. The potential of phage therapy to combat vibriosis in fish larvae production has not yet been examined. We describe the isolation and characterization of two bacteriophages capable of infecting pathogenic *Vibrio* and their application to prevent bacterial infection in fish larvae. Two groups of zebrafish larvae were infected with *V. anguillarum* (∼10^6^ CFU mL^−1^) and one was later treated with a phage lysate (∼10^8^ PFU mL^−1^). A third group was only added with phages. A fourth group received neither bacteria nor phages (fish control). Larvae mortality, after 72 h, in the infected and treated group was similar to normal levels and significantly lower than that of the infected but not treated group, indicating that phage treatment was effective. Thus, directly supplying phages to the culture water could be an effective and inexpensive approach toward reducing the negative impact of vibriosis in larviculture.

## Introduction

Aquaculture industries frequently suffer heavy financial losses that threaten their growth and sustainability, due mainly to uncontrolled microbial diseases [1], [2], [3]. Several factors may contribute to disease outbreaks, such as unfavorable environmental conditions, overfeeding, high water temperature, fast bacterial growth, infrequent water renewal, and improper removal of wounded and dead fish from the farming area.

Bacteria are the main pathogenic agents in the aquaculture industry. Vibriosis is the primary disease of marine and estuarine fish in both natural and commercial production systems throughout the world, but it may also occur in freshwater fish [4], [5], [6], [7], [8], [9]. This bacterial infection causes significant mortality in fish, up to 100% mortality in infected facilities, and is responsible for most of the current disease outbreaks in fish farming plants. Vibriosis is caused by species from the genera *Vibrio* (i.e., *V. anguillarum*, *V. vulnificus*, *V. alginolyticus*, *V. parahaemolyticus* and *V. salmonicida*) and *Photobacterium* (i.e., *P. damselae* subsp. *damselae*, formerly *Vibrio damselae*) [5], [6].

The regular use of artificial feed supplemented with antibiotics in an effort to prevent the spread of diseases and to control infections in aquaculture systems, has resulted in the development of resistant strains that render antibiotic treatments for infection ineffective. In fact, in the marine environment, most (∼90%) bacterial strains are resistant to more than one antibiotic and ∼20% are resistant to at least five antibiotics [10], [11]. The observed resistances limit the applicability of antibiotics as treatment against marine pathogens. Although commercial vaccines against vibriosis are available for fish, vaccination is not an option for fish larvae as it is unfeasible to handle large numbers of these small-sized and frail organisms. Moreover, fish larvae do not have the ability to develop specific immunity [12], which provides an added advantage of reducing the possibility of phage particle removal from the circulatory system by the host defense system [13].

Alternative strategies must be developed to control fish diseases in aquaculture. These strategies should reduce the risk of developing and spreading microbial resistance, and be reasonably inexpensive and more environmentally friendly. Phage therapy is a proven eco-friendly alternative approach to prevent and control pathogenic bacteria in aquaculture [4], [14], [15], [16], [17], [18], [19], [20]. The use of phages to prevent infection or to inactivate different fish pathogenic bacteria is well documented [15], [18], [21], [22], [23], [24], [25]. Experimental results with marine animal models have demonstrated the therapeutic efficacy of phage therapy against infectious diseases caused by *Pseudomonas aeruginosa*, *Photobacterium damselae subsp. piscicida*, *Enterococcus seriolicida*, *Aeromonas salmonicida*, *Vibrio harveyi*, *Vibrio parahaemolyticus*, *Vibrio anguillarum*, *Pseudomonas plecoglossicida*, and *Lactococcus garvieae* [4], [14], [15], [16], [17], [18], [19], [20]. Some animal models include the yellowtail (*Seriola quinqueradiata*), larval stages of shrimp (*Penaeus monodon*), Ayu (*Plecoglossus altivelis*), Atlantic salmon (*Salmo salar*), rainbow trout (*Oncorhynchus mykiss*), seabass (*Dicentrarchus labrax*), and seabream (*Sparus aurata*) [4], [14], [15], [16], [17], [18], [19], [20].

Selection of the appropriate bacteriophage, the stage of life (eggs, larvae, juveniles, or adult fish) during which phage therapy is applied and the method of phage delivery are key factors in the success of the treatment.

The success of phage therapy to control pathogenic bacteria of fish depends on virus survival in aquaculture water and their ability to inactivate a broad range of fish pathogens. The phage burst size (number of phages produce by each host cell) and the latent period (time elapsed from virus entry into the cell until the first progeny are released) are also important factors to consider when phages are selected. Phages with high burst sizes and short latent periods are more effective to inactivate bacteria; however, great burst sizes are associated with a long latent period [26] which makes the selection for phage therapy difficult.

In aquaculture, phage therapy can be applied as a preventive approach against bacterial infections during larvae production, before releasing them in the aquaculture tanks, thereby improving the overall production of adult fish and the sustainability of fish farming. During the intensive rearing of marine larvae, various forms of interactions between bacteria and biologic surfaces may occur [27], resulting in the formation of indigenous microbiota that can be beneficial or pathogenic for the animal. In aquaculture, fish larvae are maintained in incubators with hatching eggs and debris, resulting in a 1000-fold increase in bacterial counts of the culture water throughout hatching [28]. Marine fish larvae begin drinking before the yolk sac is consumed and thus bacteria enter the digestive tract before active feeding starts [27]. Older larvae may also ingest bacteria by grazing on suspended particles and egg debris [29], [30], [31].

In larval cultures, phages can be supplied in the feed, using infected bacteria as a vehicle or by direct release into the culture water [1], [15]. The use of bacteria infected with phages as carriers can be seen as a protective method to insure that phage particles are delivered directly to the organ infected without suffering any damage. However, Nakai et al (1999) demonstrated that this strategy did not enhance the protective effect. When they administrated *Lactococcus garvieae* infected with phages to treat the infection, the curative effect of the phage was not influenced, but the results did not differ from those when phages were directly administrated. The later strategy is inexpensive, flexible, and requires no specific equipment, but the antimicrobial effects are assumed to depend on phage stability in the medium and their ability to arrive at the infected tissues (i.e., intestine) by passive diffusion. Consequently, to develop an effective, safe and controlled phage therapy protocol to be used in larviculture, detailed information is needed on the properties and behavior of the selected phage. The host range of the phage, the phage time of permanence in the water, its latent period, the burst size, lytic potential, its avoidance of lysogenic induction and conversion, and the potential development of host resistance are crucial factors that must be considered.

The aim of the present study was to test the efficacy of phage therapy during the production of fish larvae (Zebrafish - *Danio rerio*) experimentally exposed to *V. anguillarum*. This bacterium was selected as a model species due to its ability to infect a large number of cultured fish species [4], [5]. A phage isolated on *V. parahaemolyticus* (VP-2) was used in the phage therapy experiments, as preliminary trials revealed that this phage was more efficiently inactivated *V. anguillarum* than the phage isolated on *V. anguillarum* (VA-1). Zebrafish was used as a biologic model system because it is a well understood, easily observable, and testable organism [32].

## Experimental Procedures

### Ethics Statement

Sewage water was collected in the EEIS9 lift station of the Aveiro Network Sewage (Portugal) with the permission of Sistema Multimunicipal de Saneamento da Ria de Aveiro (SIMRIA). (http://www.simria.pt/index.php). The field studies were done with permission of the fish farm Corte das Freiras of Aveiro (Portugal) (https://www.racius.com/corte-das-freiras-aquicultura-lda/).

The *in-vivo* studies were carried out in strict accordance with the recommendations of European Commission (2003/65/CE and 2007/526/CE) and the Spanish legislation (RD 1201/2005). Protocols were designed to comply with the European policy on the “3 Rs”(Reduce, Refine, and Replace) in animal experimentation. All of the protocols used for the challenges were supervised by a Food Research Division AZTI-Tecnalia veterinarian and approved by the ethics committee of the competent authority (Animal Experimentation Ethical Committee, Bizkaiko Foru Aldundia - Diputación Foral de Bizkaia). Embryos were collected directly from the breeding tanks shortly after fertilization and stored up to the test in optimal conditions. Larvae were not intentionally sacrificed during the experiment, and final sacrifice was done according with the standard procedures, using cold treatment.

### Bacterial Strains

Three bacterial strains, *Vibrio parahaemolyticus*, *Vibrio anguillarum*, and *Aeromonas salmonicida*, previously isolated from the aquaculture system Corte das Freiras in Ria de Aveiro (an estuarine system located in the north-western coast of Portugal - 8″44′W, 40′39′N) were used in this study [33], [34]. The other 7 strains used in this study were obtained either from the American Type Culture Collection (ATCC): *Photobacterium damselae* subsp. *damselae* (ATCC 33539), *Photobacterium damselae* subsp. *piscicida* (ATCC 29690), *Vibrio fischeri* (ATCC 49387), *Aeromonas hydrophila* (ATCC 7966), or previously isolated from Ria de Aveiro: *Pseudomonas aeruginosa*, *Pseudomonas fluorescens* and *Pseudomonas putida*, [35].

### Bacteria and Phage Culture and Titration

Except when annotated, bacteria were plated on Tryptic Soy Agar (TSA; Merck, Darmstadt, Germany) medium and incubated for 24 h at 25°C. Fresh cultures of the host strains, *Vibrio parahaemolyticus*, *Vibrio anguillarum*, were maintained on TSA at 4°C. When necessary, bacteria were grown in Tryptic Soy Broth (TSB; Merck, Darmstadt, Germany) overnight at 25°C (O.D._600_ of 0.8, corresponding to about 10^9^ CFU mL^−1^).

The phage titer was determined using the double agar layer method, with TSA as base agar and soft TSA (0.6% agarose) as soft agar. Bacteria were incubated for 12 h at 25°C. The spot test was used to study bacterial susceptibility, using TSA as the culture medium [36]. An aliquot of 30 µL of the phage lysate was placed onto the surface of the bacterial overlay and the occurrence of lytic plaques was verified after 12 h of incubation at 25°C.

### Phage Isolation and Purification

Sewage water from a lift station of the sewage network of Aveiro, Portugal (station EEIS9 of SIMRIA Multi Sanitation System of Ria de Aveiro) was filtered through 0.45 µm pore size polycarbonate membranes (Millipore, Bedford, MA, USA). The filtrate was added to double-concentrated TSB medium with 1 mL of fresh culture of the host, *V. parahaemolyticus* and *V. anguillarum*, to produce *V. parahaemolyticus* and *V. anguillarum* phages. The mixtures were incubated at 25°C for 18 h at 80 rpm, and then filtered through a 0.2 µm membrane (Millipore). Chloroform (final volume of 1%) was added to the supernatants and phage concentration was determined as described before. Plates were incubated at 25°C and examined for the presence of lytic plaques after 12 h.

One single plaque was removed from the agar, diluted in TSB, and then chloroform (final volume of 1%) was added to eliminate bacteria. The sample was centrifuged and the supernatant was used as a phage source for a second isolation procedure. Three successive single-plaque isolation cycles were performed to obtain pure phage stocks for both bacteria. All lysates were centrifuged at 10,000 g for 10 min at 4°C, to remove intact bacteria or bacterial debris. The phage stock was stored at 4°C and 1% chloroform (final volume) was added. The phage produced on *V. parahaemolyticus* was designated as VP-2 and the phage produced on *V. anguillarum* as VA-1.

### Phage Host Range Determination

Bacterial susceptibility to both bacteriophages was assayed for the 10 pathogenic bacterial strains (Table 1). The hosts tested included species from genera *Vibrio*, *Aeromonas*, *Photobacterium* and *Pseudomonas* that include the main pathogenic bacteria of fish [34], [37]. The spot test was used as an initial approach for the detection of bacterial infection [36] and the efficiency of plating was determined for the bacteria with positive spot tests (occurrence of lytic plaques) by the double-layer agar method. The plating efficiency for each host was calculated by comparison with an efficacy of 100% for the phages, VP-2 and VA-1, using *V. parahaemolyticus* and *V. anguillarum* as hosts, respectively. Three independent experiments were performed for each phage.

**Table 1:** Efficiency of plating (%) of phages VP-2 and VA-1 on different bacteria.

Bacteria	Efficacy of plating (%)	
	VP-2	VA-1
*V. parahaemolyticus*	100	73.2
*V. anguillarum*	93.4	100
*A. salmonicida*	92	88.8

### Phage Nucleic Acid Isolation

Bacteriophage lysates (10^9^ plaque forming units, PFU mL^−1^) were centrifuged 3 times at 6000 g for 10 min. The phage lysates were ultracentrifuged at 100000 g for 2 h at 10°C. Five hundred microliters of SM buffer were added to the pellet and was let to rest for 2 h. The suspension was then treated with DNase I and RNase A at 37°C for 20 min to remove any free nucleic acids contamination. Nucleic acid extraction from phage particles was performed as described by Griffiths *et al.* [38]. Extraction was performed by the addition of 0.5 mL of hexadecyltrimethylammonium bromide (Sigma Aldrich, St. Louis, MO, USA) extraction buffer and 0.5 mL of phenol-chloroform-isoamyl alcohol (25∶24∶1; pH 8.0; Sigma Aldrich) to the sample. The sample was lysed for 30 s in a FastPrep FP120 (BIO 101/Savant) at 5.5 ms^−1^ and centrifuged (16,000 g) for 5 min at 4°C. The supernatant was pipetted into a clean vial, mixed 1∶1 with chloroform-isoamyl alcohol (Sigma Aldrich) and centrifuged (16,000 g) for 5 min at 4°C. The supernatant was removed to a clean tube and the nucleic acids were precipitated with two volumes of 30% (wt/v) polyethylene glycol 6000 – 1.6 M NaCl for 2 h at 25°C. This mixture was centrifuged (18,000 g) at 4°C for 10 min, the pellet washed in ice cold 70% (v/v) ethanol, centrifuged (18.000 g) at 4°C for 10 min and then the pellet was air dried prior to re-suspension in 30 µL Tris-EDTA buffer. Nucleic acid yield was quantified in the Qubit 2.0 Fluorometer (Invitrogen, Carlsbad, CA, USA). The nucleic acid was then digested with DNase I (Ambion, Austin, TX, USA), and RNase I (Sigma Aldrich) separately, as described by the manufacturers. DNase I was inactivated by heating at 80°C for 5 min whereas RNase A was inactivated by EDTA (final concentration 20 mM) and Proteinase K (final concentration of 50 µg mL^−1^; Bioron) addition and incubation at 56°C for 1 h. Five microliters of nucleic acids of each reaction was then loaded onto a agarose gel and separated by electrophoresis (0.8% agarose gel electrophoresis at 80 V for 45 min), using XXL DNA Ladder GeneOn (25 kb DNA ladder) as marker and observed in a Molecular Imager (Chemi Doc XRS+, BioRad, Hercules, CA, USA). To determine whether phages VP-2 and VA-1 are single or double-stranded DNA viruses, 10 ng of DNA was incubated with 20 U of S1nuclease (MBI Fermentas, Portugal) at 37°C for 1 h as described by Sambrook *et al* [39]. The resulting product was electrophoresed through 0.8% agarose gel at 80 V for 40 min.

### Phage Survival Determination

Phage survival was tested in marine water collected on three different dates, in February, March, and May 2013, at the Aquaculture Corte das Freiras (Aveiro, Portugal). At each date, 50 mL of water (salinity 18–21 practical salinity units; pH 7.6–7.7) was filtered sequentially through 0.45-µm and 0.22-µm pore-size membranes (Millipore) and then sterilized in an autoclave (121°C for 20 min). Phage lysates were added (estimated final concentration 10^7^ PFU mL^−1^) and incubated at 25°C without shaking. The phage titer was determined as described above, at time zero and at intervals of 12 h until day 1, 24 h until day 5, 48 h until day 9, 72 h until day 12, 120 h until day 45, and 240 h until the end of the experiment (day 185). Three independent experiments were performed for each phage.

### One-Step Growth Assays

The one-step growth curves of phages VP-2 (on *V. parahaemolyticus* and on *V. anguillarum*) and VA-1 (on *V. anguillarum*) were performed according to the method of Almeida [40]. Briefly, mid-exponential host bacterial cultures of *V. parahaemolyticus* and *V. anguillarum* were adjusted to an optical density = 1 at 600 nm (∼10^9^ CFU mL^−1^) and 10 µL phage lysate was added to 10 mL bacterial culture (multiplicity of infection - MOI of 0.001). Phages were allowed to adsorb for 5 min at room temperature and the mixture was centrifuged (10,000 g for 5 min). The pellet was re-suspended in 10 mL TSB at 25°C and serially diluted to 10^−4^. Samples (1 mL) were obtained at 10 to 20 min intervals and the phage titration was done by the double agar layer method. The burst size (number of phages produced by infected bacteria) was determined dividing the average number of lysis plaques at the stationary phase by the average number of lysis plaques at the latent phase.

### Phage Therapy Assays

Preliminary studies of phage therapy were performed to select the best phage to inactivate *V. anguillarum*. VP-2 and VA-1 phages were tested using their natural bacteria as host, *V. parahaemolyticus* and *V. anguillarum*, respectively. The phage VP-2 was also tested on *V. anguillarum*.

#### Killing curves

Optimal MOI was determined using VP-2 phage on its natural host, *V. parahaemolyticus*. Tested MOI were 1, 10, 100, and 1000 using an overnight *V. parahaemolyticus* culture with 10^5^ CFU mL^−1^ and a set of serial dilutions of the phage lysate (10^6^ to 10^8^ PFU mL^−1^). Bacteria and phage were inoculated in TSB (final volume 50 mL) and incubated at 25°C without agitation (test samples). For each MOI, two control samples were included, the bacterial control and the phage control, respectively, without phages and without bacteria. Both controls were incubated exactly as the test samples. Aliquots were removed at the indicated sampling time (0, 2, 4, 6, 8, 10, 12, 14, 16, 18, 24, and 36 h) and bacteria and phages were counted as described before. Three independent experiments were performed for each MOI and the results were averaged.

Phage therapy experiments with phages VA-1 and VP-2 using *V. anguillarum* as the host were performed only at MOI 100 under the previously described conditions.

#### Prophage Detection in Host Bacteria Before and After Phage Therapy

To assess lysogeny among the isolated bacteria and to check for the occurrence of lysogenic induction after phage therapy, the mitomycin C (a known agent of prophage induction) test was applied. To detect if the natural bacteria can harbour prophages in their genome, fresh bacterial cultures of V. parahaemolyticus and V. anguillarum were used. To detect if phage-treated bacteria can incorporate the phages in their DNA, bacteria/phages suspensions (V. parahaemolyticus/VP-2 and V. anguillarum/VP-2 and V. anguillarum/VA-1), at a MOI of 100, were collected after 48 h of incubation at 25°C were used. The bacterial cultures were centrifuged at 12,000 g for 10 min to collect the pellet of bacteria and remove free phages. The pellets were resuspended in fresh TSB medium. Each bacterial culture was then split equally into two Eppendorf microtubes (1 ml in each): one was added of mitomycin C (final concentration, 1 µg mL^−1^, Sigma-Aldrich) and the other was used as control (no added of mitomycin C). The cultures were incubated overnight at 25°C without shaking and then centrifuged (10,000 g, 10 min) (ThermoHeraeus Pico, Hanau, Germany) Supernatants were checked for the presence of phages by the spot test. The absence of a clear zone after inducing stress indicates that the bacteria contained no prophages in their genome. Three independent assays were performed for each condition.

#### Determination of phage-resistant mutants

A *V. anguillarum* culture (final concentration 10^5^ CFU mL^−1^) and a phage lysate (final concentration 10^7^ PFU mL^−1^) were inoculated in TSB and incubated at 25°C without agitation for 24 hours (test sample). One control sample was included, the bacterial control, without phages, and was incubated exactly as the test sample. Aliquots of experimental samples and control were removed and plated by incorporation on TSA plates. Plates were incubated at 25°C for 24 h. To determine the mutation frequencies per cell per generation, ten isolated colonies were picked out from the TSA plates of the test sample, inoculated into ten tubes with TSB, grown with agitation until approximate concentration of 10^9^ CFU mL^−1^ and ten-fold serially diluted to 10^−6^. One millilitre of each dilution was plating, in triplicate, on TSA plates which were incubated at 25°C for six days (because some of the phage-resistant mutants grow very slowly). Simultaneously, an aliquot of the control sample was serially diluted to 10^−6^ and 1 mL of dilutions 10^−5^, 10^−6^ and 10^−7^ and plated, in triplicate, on TSA plates. To calculate mutation frequencies, mean numbers of mutants in one ml of test samples were divided by mean total numbers of control samples [41].

#### Phage Therapy in Zebrafish Larvae

As previously stated, all of the protocols used for the challenges were supervised by a veterinarian and approved by the ethics committee to comply with the European policy on the “3 Rs”, to obtain reliable scientific information using the lowest number of animals possible.

Zebrafish (*Danio rerio*) larvae were selected as model organisms for testing the efficacy of phage therapy against *Vibrio* infections. Due to its low salt requirement for growth and the success in previous experiences infecting zebrafish [42], *V. anguillarum* was selected as the candidate to infect zebrafish larvae. Bacteria were grown in TSB overnight and diluted 1∶5 in fresh TSB. After 30 min at 27°C, cells were centrifuged and washed twice with SEW (CaCl_2_ 66.2 mM, MgSO_4_*7H_2_O 13.4 mM, NaHCO_3_ 21 mM, KCl 3.8 mM, and NaCl 0.5%, all from Merck). The bacterial suspension was adjusted to ∼10^7^ CFU mL^−1^ with SEW. Four groups of zebrafish larvae at 5 d post-fecundation, with each group comprising 3 sets of 20 specimens, were selected by haphazard sampling for the present study (a total of 4 groups ×3 sets of 20 specimens each = 240 larvae were used). Each set of 20 specimens of the 4 groups was treated separately, corresponding to 3 independent samples per condition. The 3 sets of the first group were infected with *V. anguillarum* and treated with VP-2 phage (test group, larvae+*Vibrio*+phages), the 3 sets of the second group were infected with the bacterium but not treated with the phage (bacterium control, larvae+*Vibrio*), and the 3 sets of the third group were not infected with the bacterium but phages were added (phage control, larvae+phages). To the 3 sets of the fourth group neither bacteria nor phages were added (fish control). Each set of larvae was suspended in 3.0 mL SEW prior to inoculation (if they were inoculated). When required (test group and bacterium control), 250 µL bacterial culture was added (final concentration of 10^6^ CFU mL^−1^) and all groups were incubated at 27°C with shaking (160 rpm). One hour after infection, 200 µL phage solution of VP-2 (∼10^8^ PFU mL^−1^) was added (MOI 100, test group and phage control). Samples were incubated at 27°C with shaking (160 rpm) and samples were obtained at 1, 5, 24, 48, and 72 h post infection. Control groups were treated the same way, but instead of phage lysate, 200 µL (phage control), 250 µL (bacterium control), or 450 µL (no-treated group) of SEW were added. Fish mortality in each of the 3 sets of the 4 groups was determined by visual inspection of all fish (inspecting for dead fish) every 24 h. Bacterial and phage concentration was determined as described before.

### Statistical Analysis

Statistical analysis was performed using SPSS (SPSS 20.0 for Windows, SPSS Inc., Chicago, IL, USA). Significant differences among the different MOI tested using the phage VP-2 and the host V. *parahaemolyticus* and between both phages VA-1 and VP-2 using *V. anguillarum* as host were assessed by one-way analysis of variance model with the Bonferroni post-hoc test. The significance of differences was ascertained by comparing the results obtained in the test samples after correction with the result obtained for the corresponding control samples (difference between the concentration obtained in the control and the concentration obtained in the test sample) for the different times of each of the three independent assays. Significant differences between samples of larvae fish experiments were also determined. The groups infected with *V. anguillarum* treated with phage were compared with the non–treated infected fish (samples with bacteria but without phages) and with the controls (samples with phages but without bacteria and samples without bacteria and without phages. Normal distributions were assessed using the Kolmogorov-Smirnov test and homogeneity of variances was assessed by Levene's test. A value of p<0.05 was considered statistically significant.

## Results

### Presence of Prophages in the Bacterial Host

No phages were detected in the supernatant of cultures of *V. anguillarum* and *V. parahaemolyticus* or in the mixture of bacteria and phages after treatment with mitomycin C, demonstrating the absence of inducible prophages in the two strains. Phages presented lytic cycles with no evidence of lysogeny induction. However, more specific tests, such as search of integrase genes to evaluate the development of lysogeny are needed.

### Phage Host Range

Phage VP-2 infected *V. anguillarum* and *A. salmonicida* with an efficiency of 93% and 92% respectively, but was not effective against *A. hydrophila*, *P. damselae* subsp. *damselae*, *P. damselae* subsp. *piscicida*, *V. fischeri*, *P. aeruginosa*, *P. fluorescens* or *P. putida*. (Table 1). Phage VA-1 infected *V. parahaemolyticus* and *A. salmonicida* with an efficiency of approximately 73% and 89%, respectively, but was not effective against the other tested strains (Table 1). The lysis plaques of phages VP-2 and of VA-1 were clear with a diameter of about 2.0 mm.

### VP-2 and VA-1 Phage Characterization

#### Nucleic Acid Characterization

**Figure 1:**
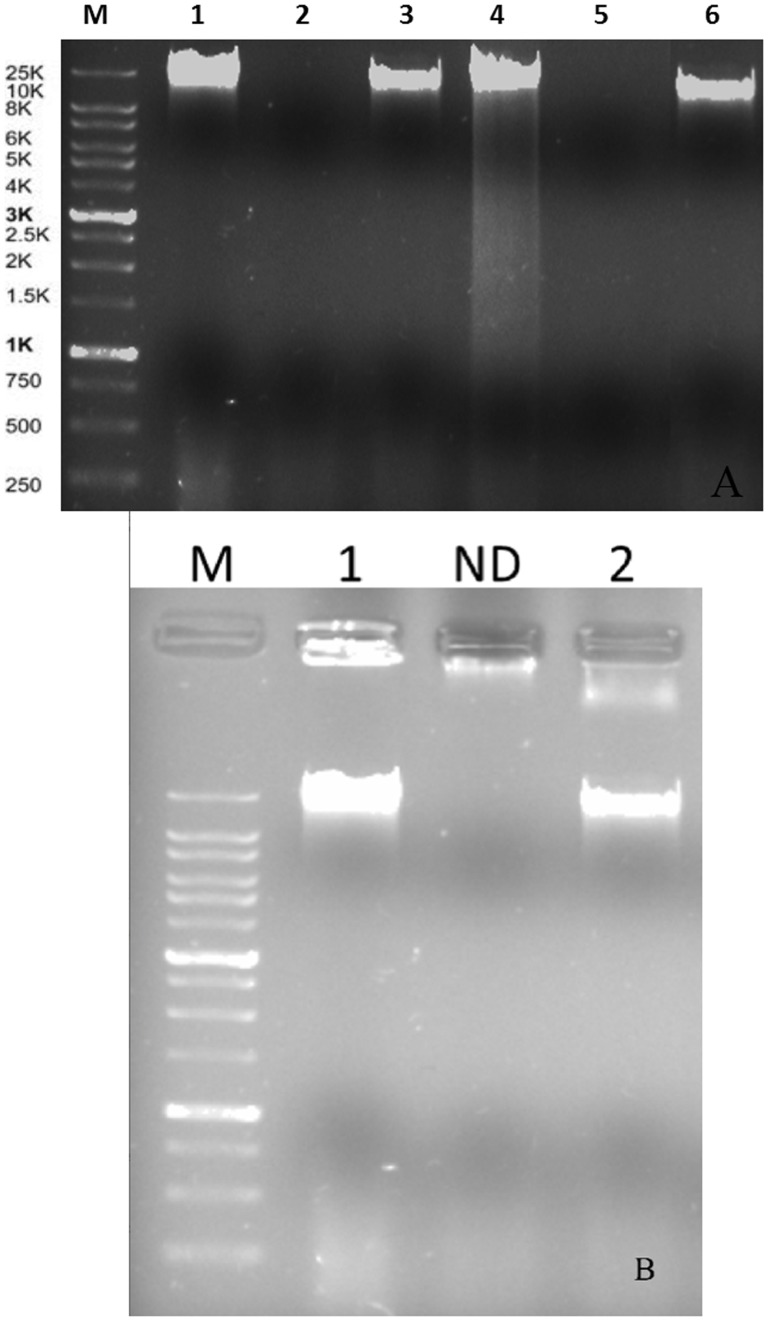
Phage DNA following digestion with DNase I and RNase A (5 µL loaded) (A), Lane M: XXL DNA Ladder GeneOn; Lane 1: uncut VP-2 phage nucleic acid; Lane 2: DNase I digested VP-2 phage nucleic acid; Lane 3: RNase A digested VP-2 phage nucleic acid; Lane 4: uncut VA-1 phage nucleic acid; Lane 5: DNase I digested VA-1 phage nucleic acid; Lane 6: RNase A digested VA-1 phage nucleic acid. Agarose gel (0.8%) showing electrophoretic patterns of S1 Nuclease restriction digestion of phage DNA(B). Lane M: XXL DNA Ladder GeneOn; Lane 1: VP-2 DNA and Lane 2: VA-1 DNA. ND – Not determined.

Nucleic acid lysates with 357 ng mL^−1^ and 210 ng mL^−1^, respectively for phages VP-2 and VA-1 were obtained. Enzyme digestion with DNase I and RNase A revealed that both phages are DNA phages (Figure 1A). Analysis of the enzyme digestion with S1 Nuclease revealed that phages VP-2 and VA-1 were double-stranded DNA viruses (Figure 1B).

#### Phage Survival

**Figure 2:**
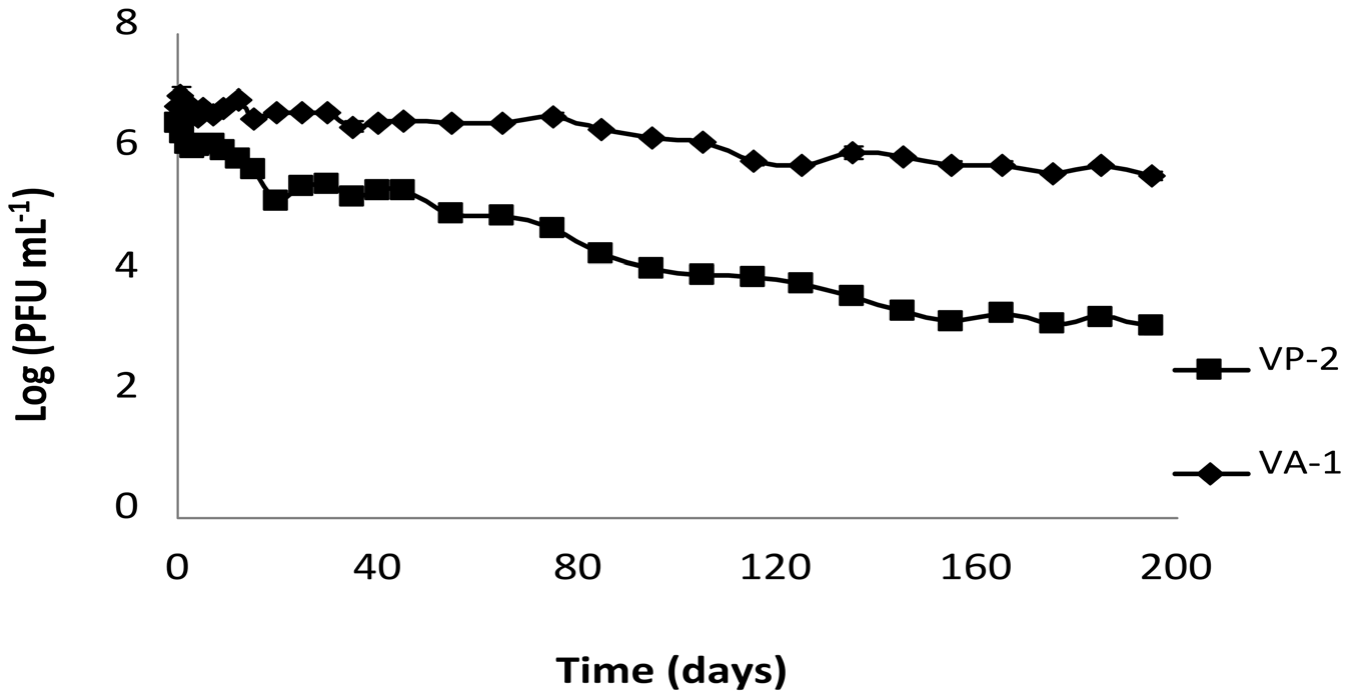
VP-2 and VA-1 phage survival in aquaculture marine water. Both phages had an initial concentration of 7 log PFU mL^−1^. Values represent the mean of three independent experiments; error bars (overlapped by symbols) represent the standard deviation.

Phage survival experiments performed in marine water from an aquaculture facility collected at three different sampling times revealed that the VP-2 phage remained viable for approximately 6 months. Phage abundance decreased by one order of magnitude on the first day, reaching a plateau over the next 12 days. Afterwards, the phage titer decreased slightly until day 35 and then more rapidly to day 195 (Figure 2).

#### Burst Size and Latent Period

**Figure 3:**
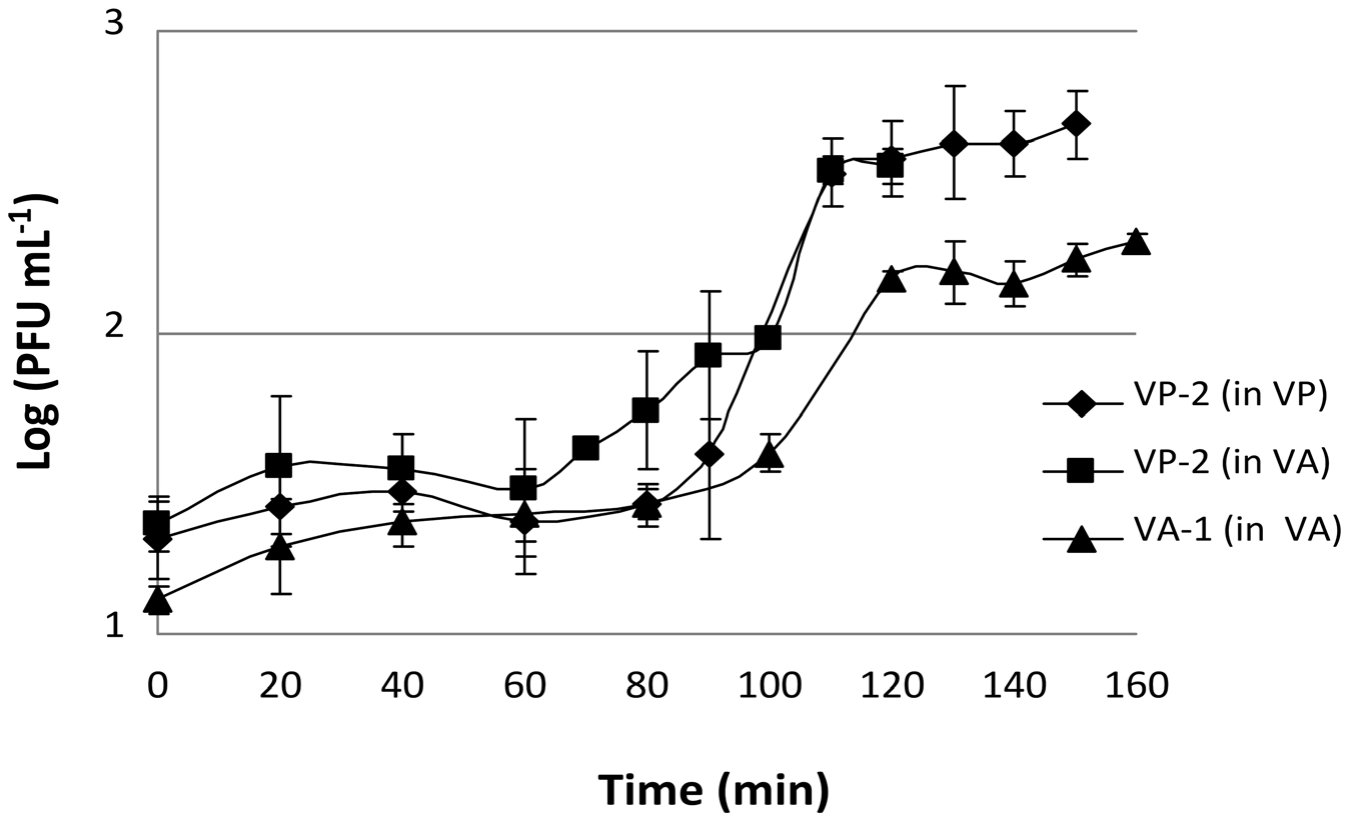
VP-2 and VA-1 phage one-step growth experiment in the presence of *V. parahaemolyticus* and *V. anguillarum* as hosts. PFU per mL are shown at different time points. Values represent the mean of three independent experiments; error bars represent the standard deviation.

The VP-2 phage one-step growth experiments revealed that lysis occurred after 90 min of incubation when using *V. parahaemolyticus* as the host. Each infected bacterium produced approximately 15 phages (Figure 3). Phage VP-2 one-step growth experiments performed using *V. anguillarum* as the phage host revealed that lysis occurred at 100 min and that each infected bacterium produced an average of 10 phages (Figure 3). When the phage one-step growth experiments were performed using the VA-1 phage on *V. anguillarum*, lysis occurred at 100 min and each infected bacterium produced an average of 6 phages (Figure 3).

### Kill Curves

#### VP-2 Phage on *V. parahaemolyticus*

**Figure 4:**
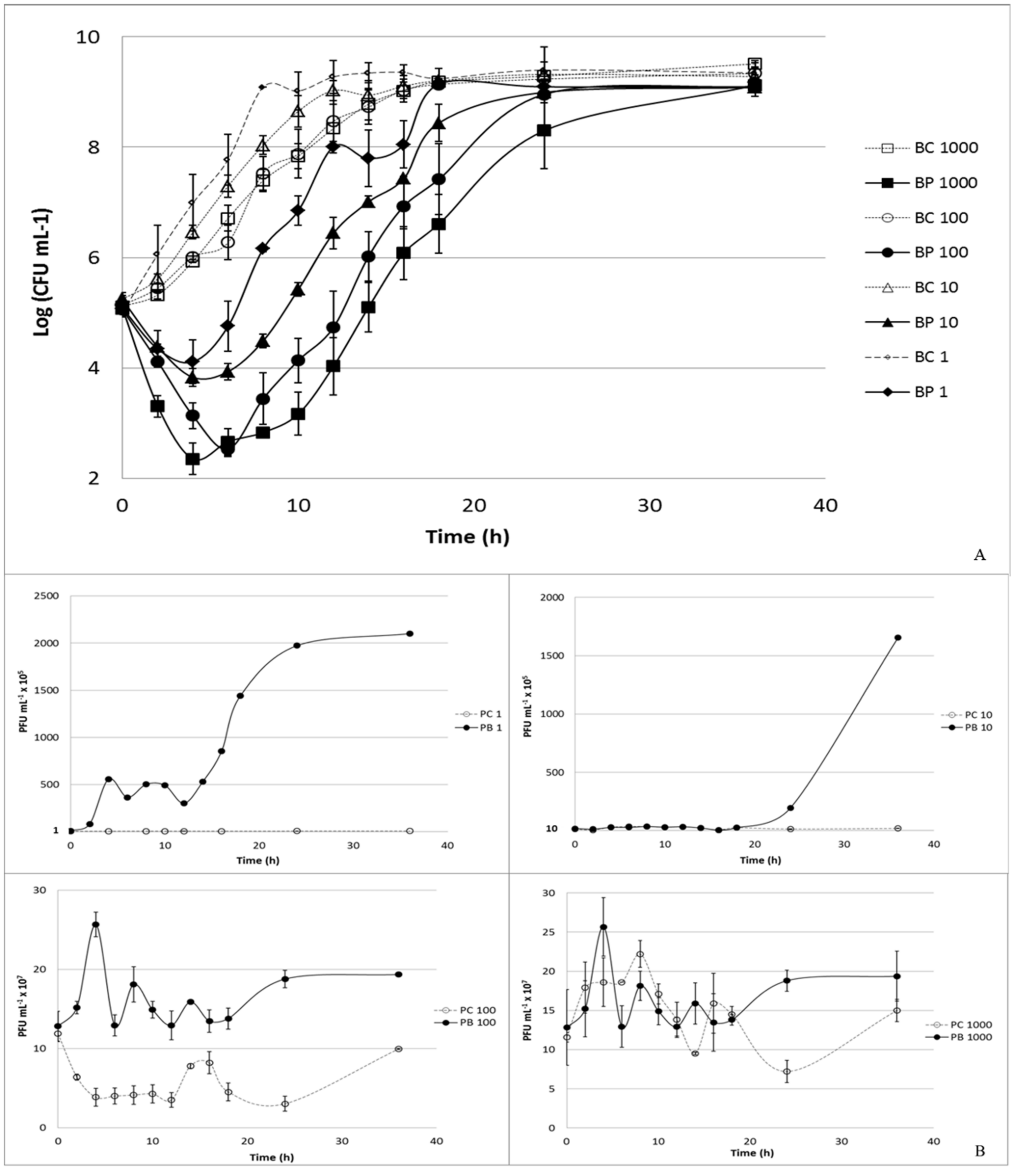
Inactivation of *V. parahaemolyticus* by the VP-2 phage at different MOI values (1, 10, 100 and 1000) during the 36 h experiment. Bacteria and phage were inoculated in TSB and incubated at 25°C. For each MOI, two control samples were included, the bacterial control and the phage control, respectively, without phages and without bacteria. Both controls were incubated exactly as the test samples. (A) Bacterial concentration (Log CFU mL^−1^): BC – Bacterial control; BP – Bacteria plus phage. (B) Phage concentration (PFU mL^−1^): PC – Phage control, and PB – Phage plus bacteria. Values represent the mean of three independent experiments; error bars represent the standard deviation.

At a multiplicity of infection (MOI) of 1, the maximum bacterial inactivation was 3.0 log colony-forming units (CFU) per mL achieved after 6 h of phage therapy. Phage therapy efficiency at MOI 1 was significantly lower than that observed at MOI 10, 100, and 1000 (p<0.05) at the different times (Figure 4A). Increasing the MOI to a value of 10 enhanced the maximum rate of inactivation to 3.6 log CFU mL^−1^ after 8 h of incubation. After 6 h, the inactivation rate was already 3.4 log CFU mL^−1^ and was, in general, significantly different from that observed for MOI 100 and 1000 (p<0.05). At a MOI of 100, the highest inactivation rate (4.1 log CFU mL^−1^) was achieved after 8 h of phage therapy, and after 6 h it was 3.7 log CFU mL^−1^. At the highest MOI value (1000), the maximum value of inactivation was 4.7 log CFU mL^−1^ after 10 h of phage therapy and 4.0 log CFU mL^−1^ after 6 h. Phage inactivation at MOI 100 was only different from that of MOI 1000 after 6 h of phage therapy (p<0.05; Figure 4A).

No decrease in phage survival was observed during the 36 h of the experiments for the VP-2 phage alone and for the phage in the presence of its host (Figure 4B). While the phage alone remained almost constant throughout (p>0.05), a significant increase (p<0.05) in 1.0 log PFU mL^−1^ was observed for the MOI of 1 (p<0.05) after 36 h when the phage was incubated in the presence of its host. Increasing the MOI to 10 also significantly increased the rate of phage survival by 0.6 log PFU mL^−1^ after the same period of time (p<0.05). For the highest MOI values (100 and 1000), only a slight increase in phage survival (less than 0.3 log PFU mL^−1^) was recorded (p>0.05; Figure 4B).

#### VP-2 Phage on *V. anguillarum*

**Figure 5:**
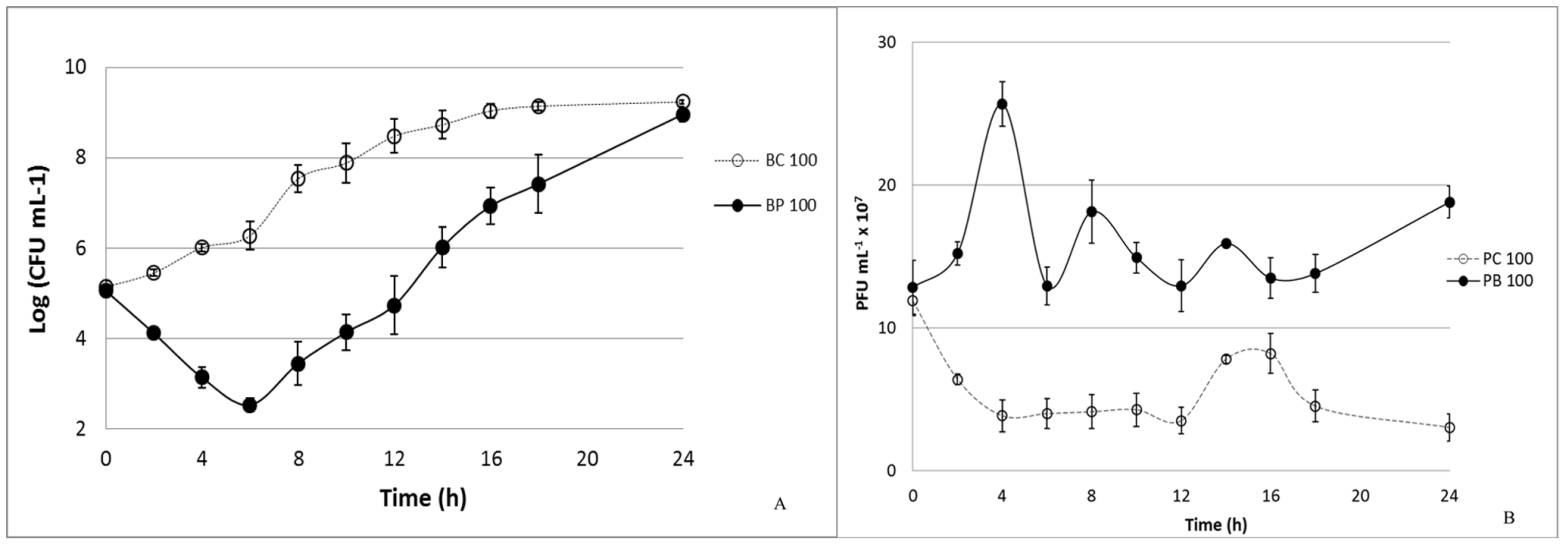
Inactivation of *V. anguillarum* by the VP-2 phage at a MOI of 100 during the 24 h experiment. Bacteria and phage were inoculated in TSB and incubated at 25°C. Phage and bacteria, had an initial concentration of 10^7^ PFU mL^−1^ and 10^5^ CFU mL^−1^. For each MOI, two control samples were included, the bacterial control and the phage control, respectively, without phages and without bacteria. Both controls were incubated exactly as the test samples. (A) Bacterial concentration (Log CFU mL^−1^): BC – Bacterial control; BP – Bacteria plus phage. (B) Phage concentration (PFU mL^−1^): PC – Phage control, and PB – Phage plus bacteria. Values represent the mean of three independent experiments; error bars represent the standard deviation.

At a MOI of 100, the maximum bacterial inactivation was 5.0 log CFU mL^−1^ achieved after 8 h of phage therapy. After 6 h of incubation, however, the rate of inactivation was already 3.8 log CFU mL^−1^ (p<0.05; Figure 5A). No decrease in phage survival was observed during the 24 h of the experiment for the VP-2 phage alone or in presence of *V. anguillarum* as the host (Figure 5B). While the phage control remained nearly constant over time (p>0.05), a significant increase (p<0.05) of 1.9 log PFU mL^−1^ was observed when the phage was incubated in the presence of its host (p<0.05; Figure 5B).

#### VA-1 Phage on *V. anguillarum*

**Figure 6:**
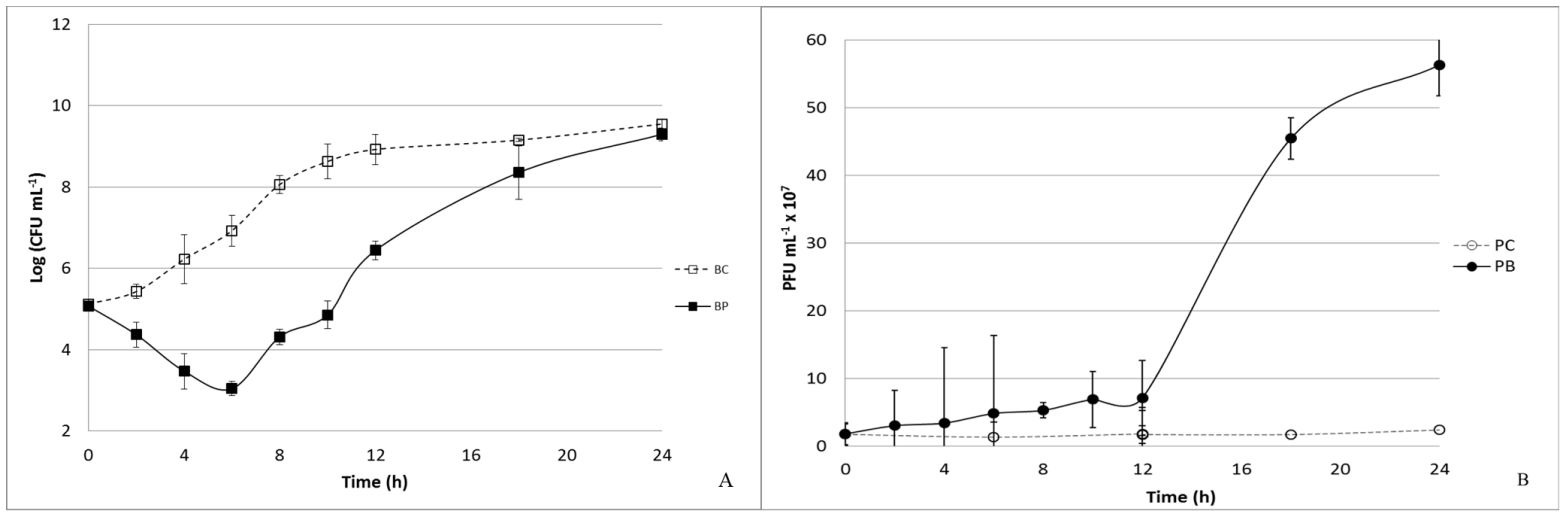
Inactivation of *V. anguillarum* by the VA-1 phage at a MOI of 100 during the 24-h experiment. Bacteria and phage were inoculated in TSB (final volume 50 mL) and incubated at 25°C. Phage and bacteria, had an initial concentration of 10^7^ PFU mL^−1^ and 10^5^ CFU mL^−1^. For each MOI, two control samples were included, the bacterial control and the phage control, respectively, without phages and without bacteria. Both controls were incubated exactly as the test samples. (A) Bacterial concentration (Log CFU mL-1): BC – Bacterial control; BP – Bacteria plus phage. (B) Phage concentration: PC – Phage control, and PB – Phage plus bacteria. Values represent the mean of three independent experiments; error bars represent the standard deviation.

The maximum rate of bacterial inactivation was 3.9 log CFU mL^−1^ achieved after 6 h of phage therapy, at a MOI of 100 (p<0.05). At 8 h of phage therapy, the inactivation rate remained quite high (2.5 log CFU mL^−1^; Figure 6A).

No decrease in phage survival occurred during the experiment for either the VA-1 phage alone or the phage in the presence of *V. anguillarum* as a host (Figure 6B). While phage control remained nearly constant during the entire testing period (p>0.05), a significant increase (p<0.05) of 1.4 log PFU mL^−1^ was observed when the phage was incubated in the presence of its host after 24 h of phage therapy (p<0.05; Figure 6B).

### Frequency of phage-resistant mutants

The frequency of emergence of resistant mutants was 8.4×10^−4^ (Table 2). Mutant colonies were visible only after 48 h but in the control sample colonies were observed after 24 h. The number of colonies after 48 and 24 h, in the sample and in the control, respectively, was constant up to 6 days.

**Table 2:** Frequencies of *V. anguillarum* spontaneous VP-2 phage-resistant mutants.

	Control sample (CFU mL^−1^)	Sample treated with phages (CFU mL^−1^)	Frequency of mutants
Days of incubation			
1	2.64±0.47×10^8^	0	0
2	2.64±0.40×10^8^	2.28±0.12×10^5^	8.40×10^−4^
3	2.64±0.40×10^8^	2.22±0.16×10^5^	8.35×10^−4^
4	2.75±0.38×10^8^	2.22±0.16×10^5^	8.20×10^−4^
5	2.99±0.74×10^8^	2.17±0.17×10^5^	8.17×10^−4^
6	2.76±0.27×10^8^	2.39±0.32×10^5^	8.11×10^−4^

### Phage Therapy in Infected Larvae

After 72 h, the group infected with *V. anguillarum* treated with phage had mortality rates similar (p>0.05) to those of fish control and the group treated with only the phage but without exposure to *Vibrio* (mean below 3%, Table 3). The mortality rate was much lower (p<0.05) than that observed for the non–treated infected fish (17%; Table 3). The larvae that were exposed to only phage were not affected during the experiment.

**Table 3:** Fish mortality after 72 h of treatment at 27°C.

Sample	Mortality
	Assay 1	Assay 2	Assay 3	Sum of all assays	Average percentage of all assays
	(n = 20)	(n = 20)	(n = 20)	(n = 60)	(n = 60)
*Vibrio*+phage	0	0	1	1	2±3^a^
*Vibrio*	3	4	3	10	17±3^a^^,^ b
Phage	0	0	0	0	0^b^
Control (non-*Vibrio* and non-phages)	0	1	1	2	3±3^b^

Values represent the Mean ± Standard Deviation of three samples.

a significant difference with Vibrio samples (samples with bacteria but without phages),

b significant difference with phage samples (with phages but without bacteria) and with control samples (non-Vibrio and non-phages) (p<0.05).

Bacterial concentrations in infected and treated larvae group samples increased until 24 h of incubation but after 48 h decreased by ∼1.5 log CFU mL^−1^. At 72 h, the number of bacteria increased, reaching values similar to those observed at the beginning of the experiment, but there was a clear shift in the colony type on the TSA medium. The colonies detected at 24, 48 and 72 h were not consistent with the *Vibrio* style colonies on TSA. The pattern of variation of the bacterial concentrations in samples corresponding to fish larvae infected with bacteria but not treated with phage was similar to that of the infected and treated samples (Table 4). The number of bacteria in the samples of the phage control group and in the fish control samples increased by 6 log CFU mL^−1^ during the experiment, reaching values similar to those obtained in the infected and treated samples and in the bacterial control samples (Table 4).

**Table 4:** Concentration (CFU mL^−1^) of viable bacteria during the 72 h experiment.

	Time (h)					
Conditions	0	1	5	24	48	72
**Vibrio+phage**	3,55±0,0×10^6^	6,69±0,76×10^6^	3,44±0,47×10^7^	8,06±1,14×10^7^	2,75±0,0×10^6^	1,55±0,52×10^6^
**Vibrio**	3,55±0,0×10^6^	6,64±1,81×10^6^	1,52±0,17×10^7^	1,46±0,32×10^7^	3,89±0,0×10^6^	1,93±1,27×10^6^
**Phage**	0	<3,16±0,0×10^2^	>1,00±0,0×10^2^	3,72±0,12×10^7^	ND	8,50±0,46×10^5^
**Fish Control (No phage or bacterial cells added**	0	<3,16±0,0×10^2^	>1,00±0,0×10^2^	3,82±0,0×10^5^	6,03±0,0×10^5^	8,82±0,43×10^5^

ND – Not determined. Values represent the Mean ± Standard Deviation of three samples.

NOTE: After 48 h, there is a shift in the major bacterial type, with the new colony types not matching *Vibrio* colonies.

## Discussion

In recent years, the potential of phage therapy to control bacterial infections in aquaculture has generated great expectations [4], [14], [15], [16], [17], [18], [19], [20]. To permit its commercial application, however, an accurate evaluation of this approach is needed for the development of effective, safe, and controlled protocols. Although studies have addressed the use of phages to control vibriosis in aquaculture [4], [5], [20], the present study is the first to examine the potential to control vibriosis in fish larvae production. The selection of the appropriate bacteriophage, the phage delivery method, and the life stage (eggs, larvae, juveniles, or adult fish) during which phage therapy is applied are key factors in the success of phage-mediated control of *Vibrio* in aquaculture.

The criteria required to select phages for phage therapy in aquaculture include i) host range; ii) latent period; iii) burst size; iv) survival in the environment; and v) efficiency of bacterial inactivation. The two dsDNA phages tested in this study (isolated on *V. parahaemolyticus* and on *V. anguillarum*): i) infect the same three hosts (*V. parahaemolyticus*, *V. anguilla*rum, and *A. salmonicida*), presenting high efficiency to inactivate the both pathogenic *Vibrio* species tested; ii) present long periods of survival in marine aquaculture water; and iii) have similar latent periods and burst sizes. Nonetheless, phage VP-2 showed a higher efficiency to inactivate *V. anguillarum* than phage VA-1 (maximum of *V. anguillarum* inactivation of 5.0 log CFU mL^−1^ for VP-2 and of 3.9 log CFU mL^−1^ for VA-1), even higher than that obtained when this phage was used to inactivate its own host (*V. parahaemolyticus*; 4.2 log CFU mL^−1^ maximum inactivation).

The results of this study strongly suggest that the VP-2 phage can protect fish larvae against *Vibrio* infections. Although infecting fish with *V. anguillarum* in the absence of the phage resulted in low fish mortality (17%), the survival of infected fish larvae in the presence of the phage increased significantly, reaching values similar to those obtained in the control treatment (fish control). Addition of the phage lysate to the fish larvae without bacterial challenge did not decrease larvae fish survival. The mortality in this group was similar to that of controls (fish control). Although phage VP-2 produced no observable effects in larval fish (morphological alterations or mortality), it is necessary to evaluate this phage (whole genome sequencing) for the presence of genes encoding toxins and antibiotic resistance. The phage lysate could also potentially contain residual bacterial antigens or endotoxins [39]. In this study, fish larvae experimentally treated only with the phage preparation (phage lysate at ∼10^9^ PFU mL-^1^ diluted 10-fold) did not show any negative effect on fish health, therefore, it is likely that the VP-2 phage lysate has few or no toxins. Moreover, as infecting fish with *V. anguillarum* in the absence of the phage resulted in low fish mortality (17%), future experiments using several concentrations of bacteria at a MOI of 100 in order to get a higher fish killing rate or to reach a plateau, would be very useful to confirm the potential of phage therapy to inactivate pathogenic bacteria in fish.

Phage VP-2 significantly improved the survival of larval fish infected with *V. anguillarum*, but killing curves assays demonstrated that, 8 h after phage addition, some bacteria remained viable and could regrow, as reported in previous studies [43], [36]. A big fraction of the remained bacteria are phage-resistant mutants (frequency of phage-resistant mutants 8.4×10^−4^) but it has been shown that virulent bacteria which become resistant to phage infection are less fit or lose their pathogenic properties [45], [46], [47]. This occurs mainly because the cell surface components, such as LPS and proteins, that act as receptors for phage adsorption also can act as virulence factors. If similar situations occur *in vivo*, it is worth mentioning that the bacterial population would assume a non-lethal state (non virulent to fish larvae). Mutations in these receptors to develop resistance to the phage would reduce pathogenicity [45], [46], [47], [48], and, in this case, bacteria regrowth after phage therapy would have few or no consequences for fish larvae. Further studies are needed to detect mutation in outer bacterial molecules of resistant bacteria after phage therapy, as these can act as phage receptors and possibly, at the same time, as virulence factors. Moreover, it was observed that during treatment the phage titer increased despite bacterial numbers have remained constant or increased, thus suggesting that cell turnover of these resistant bacteria is likely occurring to generate the phage. Future microscopic studies should look at the bacterial cells to characterize their morphological diversity, in order to understand what is occurring during phage therapy.

Levin and Bull [49] also proposed that phages decrease the bacterial level enough to be eliminated by the fish immune system by acquired response, but this is not the case in fish larvae because they are unable to develop specific acquired immunity [12]. Further studies on this topic are needed.

Whether or not it is possible to supply phages to cultured fish by intraperitoneal or intramuscular administration [9], [15], [16], research on bacteriophage treatment in aquaculture has mainly focused on oral administration [5], [9], [15], [16], [17], [18]. Oral administration can affect the phage viability due to the harsh conditions of the gastrointestinal tract [15], [16]. In fish larviculture, where mass mortality is commonly associated with the actions of opportunistic bacteria, phages cannot be supplied by oral administration and must be directly released into the culture water. Consequently, phage survival in aquaculture water must be high to reach the specific site of infection, the intestine. As the phage VP-2 is able to survive long periods in marine aquaculture water (at least 5 months), the release of the phage directly into the water allows the phage to control not only the bacteria inside the larvae fish, but also avoid colonization on fish larvae skin. In the case of infections by *V. anguillarum*, avoiding colonization on the fish skin can play a key role in preventing disease development [50]. In addition, the survival of VP-2 in aquaculture water allows for the use of phage therapy as a prophylactic measure to prevent infections, as phages can inactivate pathogenic bacteria in the water without affecting the beneficial bacterial community (e.g., probiotics). In fact, in larval fish assays, the phage VP-2 controlled *V. anguillarum* growth but did not affect other bacteria that grew during the experiment. After 24 h of treatment, the concentration of *V. anguillarum* began to decrease, but at 48 and 72 h the bacterial density increased. At 48 and 72 h, however, there was a shift in the major bacterial type, with the new colony types not matching *Vibrio* colonies. Other bacteria already present in the larvae may have grown in the saline embryo water (SEW) and masked the decrease in the *Vibrio* concentration. Actually, the growth of these bacteria was also observed in the control group with added phage but not infected with bacteria and in the control with no bacteria or phage added. The bacterial colonies that do not present the *Vibrio* type were, most likely, *Proteobacteria* associated to the zebrafish gut, faeces and mucus (e.g. *Aeromonas sp. or Pseudomonas*) [51], [52]. Although the presence of these contaminating bacteria can mask the decrease in the *Vibrio* concentration, these contaminating bacteria and other bacteria, will also be present in aquaculture water and in fish when phage therapy is applied in the field.

## Conclusions

Overall, our findings suggest that phage therapy is a suitable alternative approach against vibriosis in aquaculture, with phage administration directly to the culture water as a promising method for treatment of fish larvae. Further studies need to be performed in the field and in a real scale, at a semi-intensive fish farm, where environmental conditions are known to vary considerably at different time scales, to evaluate the potential of this approach under environmental/seasonal variation. As MOI of 10 and even of 1 are enough to efficiently inactivate the pathogenic bacteria, phage lysates with about 10^9^ PFU mL-1 can be diluted 10 to 100 times. In this way, small scale production (5 L) of phages is enough to produce sufficient material for purification. The resulting suspensions can be scaled-up, using pilot stirred bioreactors working with culture volumes of 10 to 300 L, to increase batch size. As phage solutions at 10^7^–10^8^ PFU mL-1 are enough, with 5 L of stock suspension 50–500 L of work suspension can be easily obtained. About 50 L of phage solution at 107–108 PFU mL-1 are enough for a treatment at real scale. As bacterial infection occurred at concentrations around 10^5^ CFU mL^−1^, phage suspensions with 10^7^–10^8^ PFU mL^−1^ allows the use of MOI of 100 which has been shown efficient rates of fish pathogenic bacterial inactivation in previous studies [37], [44]. This study provides the first evidence that phage therapy is a feasible alternative approach against vibriosis during fish larvae production in aquaculture systems.
